# The non-canonical Wnt receptor Ryk regulates hematopoietic stem cell repopulation in part by controlling proliferation and apoptosis

**DOI:** 10.1038/cddis.2016.380

**Published:** 2016-11-24

**Authors:** Farbod Famili, Laura Garcia Perez, Brigitta AE Naber, Jasprina N Noordermeer, Lee G Fradkin, Frank JT Staal

**Affiliations:** 1 Department of Immunohematology and Blood Transfusion, Leiden, The Netherlands; 2 Department of Molecular Cell biology, Leiden University Medical Center, Leiden, The Netherlands

## Abstract

The development of blood and immune cells requires strict control by various signaling pathways in order to regulate self-renewal, differentiation and apoptosis in stem and progenitor cells. Recent evidence indicates critical roles for the canonical and non-canonical Wnt pathways in hematopoiesis. The non-canonical Wnt pathway is important for establishment of cell polarity and cell migration and regulates apoptosis in the thymus. We here investigate the role of the non-canonical Wnt receptor Ryk in hematopoiesis and lymphoid development. We show that there are dynamic changes in Ryk expression during development and in different hematopoietic tissues. Functionally, Ryk regulates NK cell development in a temporal fashion. Moreover, Ryk-deficient mice show diminished, but not absent self-renewal of hematopoietic stem cells (HSC), via effects on mildly increased proliferation and apoptosis. Thus, Ryk deficiency in HSCs from fetal liver reduces their quiescence, leading to proliferation-induced apoptosis and decreased self-renewal.

In the bone marrow, blood cells develop from a small pool of hematopoietic stem cells (HSC).^1^ This rare population of cells is located in a specific microenvironment, the niche, and endows HSCs with the capacity to self-renew and provides signals to further differentiate HSCs into all blood cell lineages.^2^ A wide variety of signaling pathways regulate the fate of HSCs; in addition to these cells undergoing self-renewal or differentiation, they can also remain quiescent or undergo programmed cell death. These signaling pathways include Wnt, Notch, Hedgehog, BMP/SMAD, and many hematopoietic cytokines (SCF, TPO, angiopoietins).^3, 4^ Defects in these pathways are implicated in the development of bone marrow failure syndromes and hematologic malignancies.^5^

Various subpopulations that are the progeny of stem cells can migrate from BM to thymus, where they develop into the T-cell lineage.^6^ During thymic development, immature thymocytes gradually lose their proliferative and multi-lineage potential, and initiate a T-cell developmental program, a process called T-cell commitment.^7^ Early stages of T-cell development are phenotypically characterized by the absence of mature T-cell markers CD4 and CD8. These stages are therefore collectively referred to as Double Negative (DN). In mice, DN stages are subdivided into four subpopulations termed DN1: CD44+ CD25−, DN2: CD44+ CD25+, DN3: CD44− CD25+, and DN4: CD44− CD25−. Afterwards, thymocytes develop to immature single positive stage defined as CD3− CD8+ to initiate T-cell receptor (TCR) rearrangement. Thymocytes with functional TCRs develop into the next stage, double positive for CD4 and CD8, and subsequently differentiate into either mature single positive (SP) CD4 or CD8 T cells,^8^ which have different functional properties. CD4T cells provide help to other cells and CD8 T cells are cytotoxic.

In order to better understand processes that underlie the development of HSC into T cells, we and others have performed gene expression profiling of sorted subsets of HSCs, progenitor cell, and stages of T-cell differentiation.^9, 10, 11, 12^ We focused on the Wnt signaling pathway, as it is required for both self-renewal of HSCs as well as for proper T-cell development in the thymus.

Wnt signaling pathways have historically been characterized as either canonical (Wnt/*β*-catenin pathway) or non-canonical pathways.^13, 14, 15, 16, 17^ In the absence of canonical Wnt ligands and receptors, cytoplasmic levels of β-catenin are kept very low through the action of a protein complex (the so-called destruction complex) that actively targets β-catenin for degradation. Activation of the pathway by Wnt leads to inactivation of the destruction complex allowing buildup of the dephosphorylated form of *β*-catenin and its migration to the nucleus. In the nucleus, β-catenin binds to members of the TCF/LEF transcription factor family, thereby converting them from transcriptional repressors into transcriptional activators. In the non-canonical pathways, that use Ca^2+^ signals or JNK kinases, there are no increases in *β*-catenin levels but cell polarity or motility, as well as regulation of apoptosis are the main biological effects.

We previously observed that Wnt ligands and receptors are very dynamically expressed in HSCs, progenitors, and thymocytes.^18^ One of these genes encodes the non-canonical Wnt receptor Ryk. The Ryk (related to receptor tyrosine kinase), Ror (RTK-like orphan receptor), and MuSK (muscle-specific kinase) families of RTKs, which all have unexpected links to Wnt signaling, probably use a unique activation mechanism.^19^ Ryk contains a Wnt-inhibitory factor-1 domain in its extracellular region and was hypothesized to function as a receptor (or co-receptor) for Wnts, but lacks endogenous PTK activity owing to mutations in the kinase domain.^20^ The first function of Ryk was uncovered in a screen for genes involved in Drosophila CNS axon pathfinding^21^ and as a gene required for learning and memory in flies. A further breakthrough in understanding Ryk function came from the finding that Ryk is a axon-repulsive receptor for the WNT5 protein.^22^ Most of the fly and mammalian Ryk studies to date have focused on its role in aspects of the developing or regenerating nervous system (reviewed in ref. 23), although the Ryks also have roles in other tissues. Ryk directly binds Wnt-1 and Wnt3a via its WIF domain and forms a ternary complex with Frizzled required for the induction neurite outgrowth in dorsal root ganglia explants.^24^ The intracellular domain of Ryk binds to disheveled, which is required for TCF activation in response to Wnt3a activation.

There is only a handful of studies focused on the role of Ryk in hematopoiesis and thymopoiesis. More than 20 years ago, expression analyses revealed that Ryk is regulated during hematopoietic development and stages of maturation.^25^ More recently, it was proposed that Wnt5a regulates HSC quiescence and hematopoietic repopulation through the Ryk receptor and that this process is mediated by suppression of reactive oxygen species.^26^ We recently showed that canonical and non-canonical Wnt signaling have vastly different and contrasting roles in hematopoiesis and thymopoiesis, in part, by regulating cell survival and apoptosis.^27^

None of the few previous studies on Ryk function in hematopoiesis employed genetic loss-of-function models, hence we explored the role of RYK1 using mice, which have a targeted mutation in Ryk1 generated by knocking a lacZ allele into the coding region.^28^ We analyzed the role of Ryk in four different experimental setups; (a) *ex vivo* functional gene expression analyses in neonatal mice and embryos, (b) *in vitro* assays for T-cell development in presence of the prototypical canonical and non-canonical Wnt ligands, Wnt3a, and Wnt5a, respectively,^27, 29^ (c) primary *in vivo* murine bone marrow transplantation assays (for blood cell reconstitution), and (d) secondary transplantation reconstitution assays to address self-renewal. Only subtle differences between the Ryk mutant and controls were observed in the first three assays. However, the secondary transplantation assay revealed that lack of Ryk results in lower stem cell repopulation indicating a role for Ryk in stem cell self-renewal. Our studies indicate that this is likely due to the fact that Ryk knock-out (KO) stem cells have diminished quiescence, leading to proliferation-induced apoptosis and decreased self-renewal.

## Results

**Figure 1:**
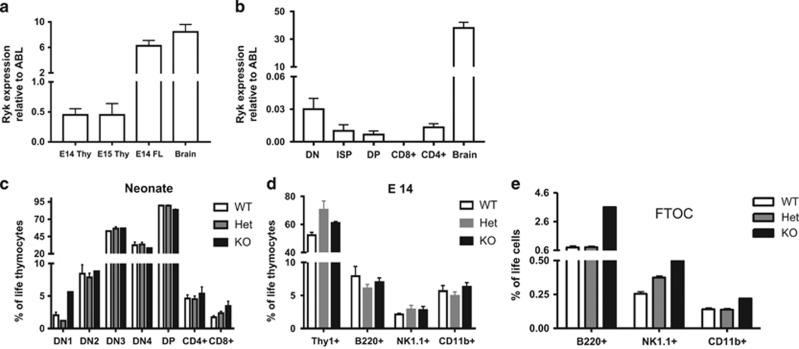
Gene expression analysis of Ryk in the murine hematopoietic system. RTq-PCR analysis was performed to determine the level of Ryk expression normalized to ABL-2 expression as a housekeeping gene. The level of Ryk expression assessed in the thymic lobes and fetal liver E14 embryos (**a**) and adult T-cell developmental subsets in the thymus (**b**). Brain tissue was used as a positive control. Data are mean±S.D. of six mice. Flow cytometric analysis performed in E14 thymocytes (**c**) and T-cell developmental subsets of neonates (**d**) in Ryk WT, Het, and KO mice. Data are mean±S.D. of three mice per group (**e**) Frequency of non-T-cell lineages in cultured thymic lobes in fetal thymic organ cultures

In order to assess gene expression patterns of Ryk in the murine hematopoietic systems, in particular during T-cell development, quantitative PCR was performed. First, we quantified Ryk expression in embryonic thymic lobes and fetal livers (FLs). Brain tissues were used as a positive control, as brain provides a rich source of Wnts and their receptors. The expression of Ryk was ~12-fold higher in FL, the site of hematopoiesis in the embryo, relative to the thymic lobes (Figure 1a). We also quantified Ryk expression during T-cell developmental stages in the adult murine thymus. The overall level of Ryk expression was much lower in the adult thymus compared with the embryonic thymic lobes. Nevertheless, the highest level of Ryk expression was observed at the most immature stage of DNs, and declined as thymocytes developed further. Notably, SP CD4+ T cells had a relatively higher Ryk expression compared with the SP CD8+ T cells (Figure 1b).

Because this spatially and developmentally regulated expression pattern suggested a potential functional the role for Ryk in thymopoiesis, we assessed thymic T-cell development phenotypically in Ryk-deficient neonates. However, no differences were observed in the T-cell developmental stages when we compared Ryk WT with Ryk/+ and Ryk −/− thymi in neonates (Figure 1c). We also looked into the thymocyte subsets in E14 thymic lobes and again no difference was detected among different Ryk genotypes (Figure 1d). Yet, when thymic lobes were cultured *in vitro,* an increase in NK cells was observed.

**Figure 2:**
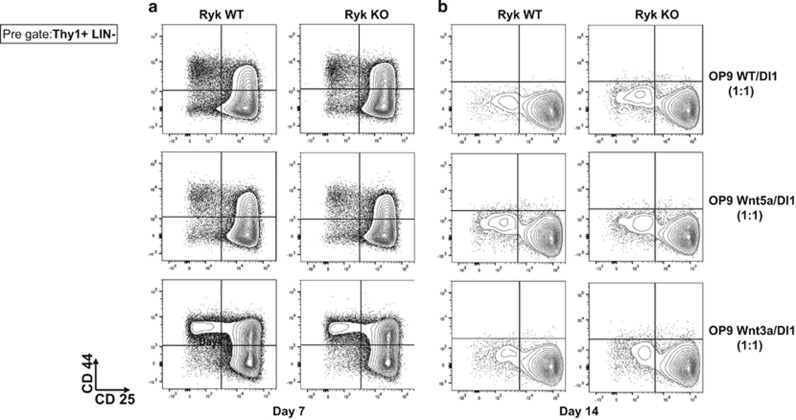
Phenotypic analysis of Ryk KO T cells developed *in vitro* in presence of prototype Wnt3a and Wnt5a. E14 FL cells were obtained from Ryk WT and Ryk KO embryos and were co-cultured with mixture of OP9WT/DL1, OP9 Wnt3A/DL1, and OP9 Wnt5A/DL1 1:1. Cells were harvested 7 days (**a**) and 14 days (**b**) after co-culture and were analyzed flow cytometric for DN stages of T-cell development. The plots are pre-gated for Thy1+ and LIN− markers. The representative plots of two independent experiments are shown. Three mice per experiment were used

It is known that Ryk can act as a co-receptor to induce Wnt signaling.^30^ In fact, it has shown that Ryk can bind to both Wnt3a and Wnt5a, and trigger canonical and non-canonical Wnt signaling pathways, respectively.^24, 30, 31^ To investigate the effects of Wnt signaling together with Ryk during T-cell development, we performed an *in vitro* T-cell development assay using E14 FL cells as a source of HSCs. In order to support multi-lineage differentiation, we mixed OP9 WT with OP9 DL1 (which induces T-cell development) in a 1:1 ratio as a control, and compared that with OP9 Wnt5a/DL1 1:1 and OP9 Wnt3a/DL1 1:1. T-cell development was assessed phenotypically at Day 7 and Day 14 post co-culture of FL cells with OP9 cells. No difference was observed between Ryk WT and Ryk KO FL cells (Figures 2a and b). We also examined NK cells, early B cells and myeloid cells but no significant phenotypic differences were detected between Ryk WT and Ryk KO FL cells in any of the conditions (data not shown).

**Figure 3:**
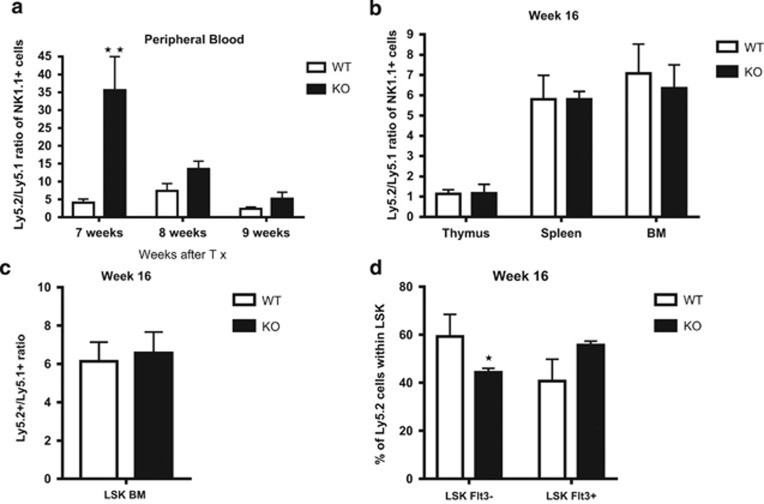
Peripheral NK cells analysis in recipient mice reconstituted by Ryk-deficient LSKs. Lin− Sca1+ Kit+ (LSK) cells were sorted from E14 FL Ryk WT and Ryk KO embryos and were transplanted intravenously into the Ly5.1-irradiated recipients. Peripheral blood analysis performed at 7, 8, and 9 weeks post transplantation. (**a**) The ratio of Ly5.2/Ly5.1 NK1.1+ CD3− cells in the recipient mice reconstituted with Ryk WT and Ryk KO LSKs is depicted. The recipients were killed 16 weeks after transplantation and the thymus, spleen, and BM were analyzed for NK cells. (**b**) The ratio of Ly5.2/Ly5.1 of NK1.1+ CD3− cells is depicted. The Ly5.2/Ly5.1 ratio of LSK compartments in the BM of recipient mice after 16 weeks of transplantation is shown (**c** and **d**). Data are mean±S.D. of five mice per group. *P<0.05 and **P<0.01

In order to investigate the effect of Ryk deficiency during hematopoiesis and lymphopoiesis *in vivo* we performed competitive murine reconstitution assays. In such assays, the test and competitor population differ only in alleles of CD45 namely, CD45.1 (Ly5.1 historically) and CD45.2 (Ly5.2 historically). These two alleles are believed to be functionally identical, but can be readily discriminated by antibodies and allow for tracking of various cell populations and their progeny. LSK cells sorted from E14 FL Ryk WT or Ryk KO that bear the Ly5.2 congenic marker were mixed in a 1:1 ratio with WT LSKs from Ly5.1 congenic background. Next, the mixture of cells was transplanted into irradiated Ly5.1 recipients. A marked increase in CD3− NK1.1+ peripheral NK cells was observed in the recipients reconstituted by Ryk KO LSKs at week 7 post transplantation (Figure 3a). To follow-up on these observations, we analyzed the recipients every week. However, the phenotype was not observed at later time points (only at week 7 and 8 with a slight increase at week 9). At the end of the experiment we thoroughly examined NK cell development in the thymus, spleen and BM of the recipients and did not observe any differences between Ryk WT and Ryk KO recipients (Figure 3b). We also analyzed the stem cell reconstitution and LSK compartments in the BM of recipients. The Ly5.2 to Ly5.1 ratio of LSK (Figure 3c), LSK Flt3−, and LSK Flt3+ (Figure 3d) were not altered in the Ryk-deficient chimeric BMs. However, when thymic lobes were cultured *in vitro,* in a so-called fetal thymic organ culture, an increase in NK cells was observed consistent with the temporal increase in NK cells observed after transplantation. Hence, the mild phenotypic difference in NK cells may result from the mild block at DN stages, where NK cells in the thymus split off from the T-cell lineage.

**Figure 4:**
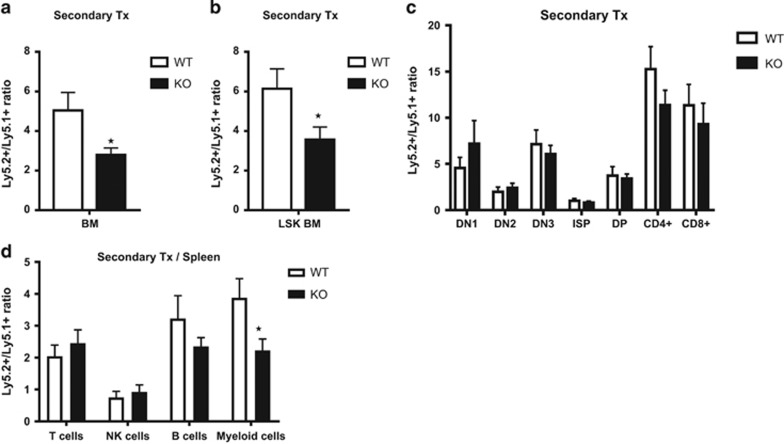
LSK and T-cell development analysis in secondary recipient. Primary BMs were obtained from the recipients reconstituted with Ryk WT and Ryk KO LSKs and transplanted into the L5.1-irradiated secondary recipient. Twelve weeks after transplantation, the secondary recipients were killed and BM and thymus were analyzed by flow cytometry. The ratio of Ly5.2/Ly5.1 (**a**) BM LSKs (**b**) and T-cell developmental subsets in the thymus (**c**) are depicted. Data are mean±S.D. of four mice per group. *P<0.05

Although, no significant alteration was observed owing to Ryk deficiency at the level of stem cells in BM, there seemed to be a trend of fewer stem cells in Ryk KO BMs (Figure 3d). As we detected high levels of Ryk gene expression in FL cells and most immature thymocytes, we hypothesized that Ryk might be involved in stem cell repopulation or self-renewal of HSCs. To further investigate this hypothesis, we performed secondary transplantation assay with chimeric BMs obtained from the primary recipients. This assay is the gold standard for investigating self-renewal properties of HSCs.^32^ Three out of four mice reconstituted with Ryk KO primary chimeric BM showed lower reconstitution of Ly5.2+ cells compared with the recipients reconstituted with Ryk WT primary BMs (Figure 4a). Further analysis revealed that lower reconstitution in Ryk KO group was caused by fewer Ly5.2+ LSK cells relative to Ryk WT group (Figure 4b). In contrast to the stem cell-mediated phenotype, no significant differences were observed during T-cell developmental stages in the thymus (Figure 4c).

**Figure 5:**
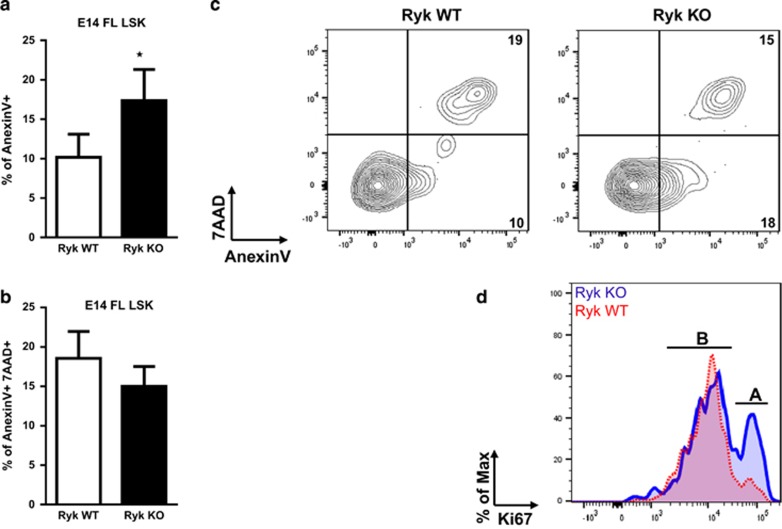
Apoptosis and proliferation analysis of E14 FL LSKs *ex vivo.* The percentage of AnexinV+ apoptotic cells (**a**) and AnexinV+ 7AAD+ dead cells (**b**) of Ryk KO and Ryk WT E14 FL LSKs are depicted. The representative plot of three mice per group is shown (**c**). The proliferation status of cells was assessed by Ki67 staining. The representative plot of three mice per group is shown (**d**). Data are mean±S.D. of three mice per group. *P<0.05

To study the mechanisms underlying reduced stem cell repopulation in the absence of Ryk, we performed apoptosis and proliferation analyses. E14 FL cells were obtained from Ryk WT and Ryk KO embryos, stained for LSK *ex vivo,* and the apoptosis and proliferation status of the cells was analyzed by flow cytometry. FL LSKs derived from Ryk KO embryos were more apoptotic showed by higher percentage of AnnexinV compared with the Ryk WT LSK cells (Figures 5a and c). However, the percentage of AnnexinV+ 7AAD+ dead cells were not altered in the Ryk KO LSK cells (Figures 5b and c). In addition, proliferation analysis of FL LSKs using the Ki67 marker revealed that the percentage of proliferative LSK cells is around twofold higher in Ryk KO FLs compared with the controls. (Figure 5d). This was confirmed using cell cycle analysis on FLs from Ryk KO and wt littermate controls (Table 1) showing that wild-type cells are much more in G1 (resting) than Ryk-deficient stem/progenitor cells, where almost twice as many cells are actively cycling indicating a loss of quiescence. Thus, a combined increase in apoptosis and proliferation explains the lower self-renewal of Ryk-deficient LSK cells.

## Discussion

In this study we used a full Ryk loss-of-function model for the first time to investigate the role of this non-canonical Wnt receptor during hematopoiesis and lymphopoiesis. The model represents the null allele of Ryk generated by homologous recombination in which 14.5 kb of genomic DNA including exons encoding >95% of the Ryk extracellular domain and the entire transmembrane domain are deleted.^28, 33^ Our data suggest that Ryk as a co-receptor has a marginal role in hematopoiesis, possibly owing to a redundant role with other tyrosine kinase receptors including Ror,^34, 35^ or Wnt signaling receptors such as FRZ 2,^30, 36^ and or FRZ8 in combination with Flamingo.^37^ During neurogenesis, Ryk's function is vital, which is consistent with its high level of gene expression in neural tissues.^36^ The importance of Ryk during embryogenesis is well studied.^23^ ^38^ Indeed, we also observed that in hematopoietic tissues the level of Ryk expression is higher in FL or fetal thymic lobes compared with the adult tissues (Figure 1).

It has previously been shown that Ryk's function, similar to other receptors, is context and tissue dependent.^23, 34^ Most probably, this is determined by several factors, including abundancy of specific triggers in various tissues, or the expression of distinct co-receptors by neighboring cells. Ryk can bind to both the Wnt3a canonical ligand,^24^ and Wnt5a non-canonical ligand^26^ depending on the context and type of tissue, suggesting that different experimental settings might result in different outcomes. Our data do not support an important specific role for Ryk during T-cell development as no differences between mutant and control were observed *in vitro* in presence of both the Wnt3a and Wnt5a ligands.

We showed that Ryk has a role in stem cell repopulation when we performed secondary transplantations. Ryk KO stem cells undergo more apoptosis and are more proliferative compared with wild-type cells. Nemeth and co-workers have proposed that Ryk, by binding Wnt5a, can suppress proliferation of LSK cells.^25, 39^ In these studies on Ryk's function in hematopoiesis, anti-Ryk polyclonal antibodies that presumably block the receptor have been employed.^24, 26^ These investigators showed that by adding polyclonal antibodies to the Ryk receptor a modest decrease in cells in G0 (from 29 to 22%) was observed, which was interpreted as a loss-of-function effect, in which blocking Ryk would increase proliferation; this in line with our observation on genetically deficient Ryk stem cells. However, polyclonal antibodies have variable effects and could include both stimulating and inhibitory antibodies. In addition, structural and functional studies have shown high levels of redundancy between Ryk and other members of tyrosine kinase receptors, in particular the ROR non-canonical Wnt receptors, making the study of each receptor specifically cumbersome.^34^ Given these structural similarities, it is even possible that an anti-Ryk polyclonal antibody could cross react with other non-canonical receptors. Thus, it is uncertain if all effects attributed to blocking Ryk, could be assigned to Ryk, Ror or perhaps other receptors. A clear loss-of-function model as we employed here allows a more direct interpretation of the role of Ryk in hematopoiesis, although molecular redundancy by the related Ror receptors could also play a role here. The effects of treatment with polyclonal Ryk antibodies on long-term hematopoietic reconstitution were similar to ours results, that is, lower reconstitution when Ryk's function was lost. We have chosen to use competitive transplantation, as this reduces mouse to mouse variability and possible effects of an antibody treatment on non-hematopoietic cells can be excluded, for instance on niche cells that express Ryk. Using this system, we here provide definitive proof for Ryk's functional role in HSC self-renewal via competitive secondary transplantation, the gold standard assay to assess HSC self-renewal. Thus, Ryk deficiency in HSCs reduces their quiescence, leading to proliferation-induced apoptosis and decreased self-renewal.

Our data also suggest that a timing–dependent role for Ryk in hematopoietic tissues. We observed that peripheral NK cells are temporary higher in initial assays of recipients transplanted with Ryk KO FL cells compared with wild-type group. One possible explanation is that Ryk is only important in a certain stage of NK cell development, as a default pathway during thymic T-cell development when TCR rearrangements cannot successfully be accomplished and multipotent cells choose a NK cell fate. As the cells pass that specific stage and a critical number of T cells have been generated, the role of Ryk would become less important.

Concluding, besides a developmental window of time for NK lineage development, the effects of Ryk on thymopoiesis are apparently limited. The combined increases in apoptosis and loss of quiescence in HSCs, likely underlie the lower self-renewal of Ryk-deficient LSK cells. The roles of canonical and non-canonical Wnts and potential cross-talk between the pathways, clearly require more research, particularly, as Wnts are being employed in stem cell expansion protocols,^40, 41^ including those employing designer nucleases for therapeutic gene editing.^42^ Finally, increasing evidence indicates the involvement of both canonical and non-canonical Wnts in hematological malignancies (e.g., reviewed in ref. 13). As we showed previously, the dosage of canonical Wnt signaling is critical in determining the functional outcome on hematopoietic cells^43, 44^ and investigating Wnt proteins for HSC expansion will require good *in vivo* reporter systems and well-characterized reagents to take effects on apoptosis as well as on cell proliferation into account. Collectively, such tools would help capitalizing on the inherent power of the canonical and non-canonical Wnt pathway to regulate apoptosis and self-renewal of stem cells.

## Materials and Methods

### Mice

Mice were bred and maintained in the animal facilities of Leiden University Medical Centre, in accordance with legal regulations in The Netherlands and with the approval of the Dutch animal ethical committee. C57Bl/6-CD45.1 (Ly5.1) and C57Bl/6-CD45.2 (Ly5.2) mice were obtained from the Jackson Laboratory, and Ryk KO mice were kindly provided by Dr. S Stacker.^28^

### Flow cytometry

The following antibodies were obtained from BD Biosciences (San Diego, CA, USA): anti-CD3-APC (145-2C11), anti-cKit-Pe-Cy7 (2B8), and anti CD11b-PE (M1/70). For Lineage depletion these markers were used: CD3 (145-2C11), CD4 (L3T4), CD8 (53-6.7), CD11b (M1/70), Gr1 (RB6-8C5), B220 (Ra3-6B2), Ter119 (Ly76) and Nk1.1 (PK136) biotin and subsequently were stained with streptavidin eFluor 450 (48-4317) from eBioscience (Vienna, Austria). The following antibodies were also purchased from eBioscience: Ly5.1-PE-Cy7 (A20), Ly5.2 Alexa Fluor 780 (104), B220 PE-Cy7 (RA3-6B2), Gr1 eFluor 450 (RB6-8C5) and Sca1 PE-Cy7 (D7). Cells were stained in fluorescence activated cell sorter (FACS) buffer (PBS, 2% bovine serum albumin, 0.1% sodium azide) for 30 min at 4 °C. Ultimately, cells were washed and measured either on a Canto I, or an Aria (BD Biosciences) FACS. For FL LSK, Mac1 was precluded from the lineage gate, as FL LSK express Mac1.^45^ For apoptosis analysis, E14 FL cells were stained with 7AAD/AnnexinV kit (BD Bioscience) in combination with LSK staining. For proliferation analysis E14 FL cells were stained with PE mouse anti-Ki67 set (BD Pharmingen, San Diego, CA, USA) in combination with LSK staining or for cell cycle analysis with an adapted protocol for combined LSK and propidium iodide staining.^46^ Data were analyzed using FlowJo software (Tree Star, Ashland, OR, USA).

### Co-culture of FL cells with OP9 cell lines and fetal thymic organ cultures (FTOC)

In total, 50 000 total FL cells were obtained from Ryk WT and Ryk KO mice and were co-cultured on confluent layers of OP9 WT/DL1, OP9 Wnt3a/DL1, or OP9 Wnt5a/DL1 mixed in a 1:1 ratio as described previously^27^ with Alpha MEM 10% FCS containing 50 ng/ml rmSCF, 10 ng/ml rmFlt3L and 10 ng/ml rmIL-7 (all cytokines from R&D Systems, Abinsdon, UK) in 24-well plates. Cells were harvested after 7 and 14 days of co-culture and assessed for T-cell development by flow cytometric analysis. FTOC were done as described before^47^ using fetal thymic lobes from E14 embryos, which were genotyped for the status of the Ryk deficiency. Thymic lobes were cultured on a nitrocellulose filter on air/medium interphase for 7–14 days, dispersed, and analyzed by flow cytometry.

### Ryk gene expression analysis

Total RNA was extracted using Qiagen RNeasy mini or micro columns. One mirogram of total RNA was used as a template for cDNA synthesis, using Superscript III reverse transcriptase (Invitrogen, Carlsbad, CA, USA), Oligo dT, and random hexamer primers. The RT-PCR reaction was performed using TaqMan Universal Mastermix (Applied Biosystems, Foster City, CA, USA) and was run on a PRISM 7700 sequence detection system containing a 96-well thermal cycler (Applied Biosystems). The following primers were used in combination with FAM-labeled probes from the universal probe library (Roche, Almere, Netherlands): mouse Ryk forward primer: 5′-CAAGCTTCGAGGTCTGCAC-3′ reverse primer: 5′-ACCATGGGCTTTTCTCCTTC-3′. RQ-PCR results were normalized to Abl expression in the same sample: forward primer: 5′-TGGAGATAACACTCTAAGCATAACTAAAGGT-3′ reverse primer: 5′-GATGTAGTTGCTTGGGACCCA-3′ and probe: 5′-FAM-CCATTTTTGGTTTGGGCTTCACACCATT- NFQ-3′.

### Competitive transplantation assay

Primary transplantation assays were performed with the Ly5.1/Ly5.2 system. LSK cells were sorted from Ryk WT and Ryk KO (Ly5.2 background) and Ly5.1 WT FLs. In total, 2 × 10^3^ Ryk WT or Ryk KO LSKS were mixed 1:1 with Ly5.1 WT LSKs, and were transplanted intravenously into lethally irradiated (8 Gy) Ly5.1 (9–12 weeks) mice together with 3 × 10^5^ Ly5.1 splenic support cells. Chimeras were analyzed at 4, 8, and 12 weeks after transplantation in peripheral blood, and mice were killed for analysis at 16 weeks post transplantation except where otherwise indicated. Mice were considered repopulated when>1% multi-lineage Ly5.2 cells could be detected in nucleated peripheral blood cells 3 months after transplantation. For secondary transplantation, equal numbers of total BM cells from primary recipients that received Ryk WT or Ryk KO LSKs were pooled and transplanted into lethally irradiated Ly5.1 secondary recipients. Peripheral blood from secondary transplanted mice was analyzed at 4, 8, and 12 weeks post transplantation. Thirteen weeks after transplantation mice were killed and BM, thymus, and spleen were analyzed.

### Statistical analysis

Statistical analysis was performed using the Mann–Whitney *U*-test (Prism GraphPad Software, San Diego, CA, USA). *P*<0.05 was considered statistically significant.

## Figures and Tables

**Table 1:** Cell cycle analysis of Ryk-deficient vs wild-type fetal liver stem/progenitor cells

*Genotype*	*G1 (%)*	*S (%)*	*G2/M (%)*	*Resting/cycling*
wt	41	48	12	0.68
wt	39	48	15	0.62
Ryk−/−	23	56	20	0.30
Ryk−/−	22	60	14	0.29
